# Editorial: Sensory Adaptation

**DOI:** 10.3389/fnsys.2021.809000

**Published:** 2021-12-08

**Authors:** Mehdi Adibi, Davide Zoccolan, Colin W. G. Clifford

**Affiliations:** ^1^Neurodigit Lab, Department of Physiology, Biomedicine Discovery Institute, Monash University, Clayton, VIC, Australia; ^2^Visual Neuroscience Lab, International School for Advanced Studies (SISSA), Trieste, Italy; ^3^School of Psychology, University of New South Wales, Sydney, NSW, Australia

**Keywords:** context, neuronal adaptation, neural network, salience detection, aftereffect, perceptual adaptation, sensory system, face adaptation

In the natural word, organisms are constantly exposed to continuous streams of sensory input. These inputs are dynamic and can undergo major changes in level, at times involving over 100-fold variations in the range of the physical parameters describing the sensory *context* surrounding us. In other instances, the overall strength of the sensory signal (e.g., the luminance or contrast of a visual pattern) may remain roughly constant but the quality of the perceptual features contained in the signal may vary dramatically (e.g., a face changing its expression). In both cases, sensory neurons can adjust their sensitivity based on recent stimulus history, a process known as *sensory adaptation neuronal adaptation*. Neuronal adaptation is a universal phenomenon across sensory modalities and occurs at multiple stages of processing (Solomon and Kohn, 2014; Whitmire and Stanley, 2016). Despite the wealth of research on sensory adaptation in psychophysics and physiology, its possible functions and underlying neuronal mechanisms remain debated. The present Research Topic provides a multidisciplinary survey of recent research aimed at better understanding how the brain functions adaptively to make sense of its surrounding environment. The experimental approaches covered by this Research Topic range from human electrophysiology, functional brain imaging and psychophysics to *in vivo* and *in vitro* whole-cell and extracellular electrophysiology, histology and pharmacological manipulations in a broad range of model systems (from insects to birds, rodents and cats), attesting to the breadth of current research on this topic.

What modulatory effects of adaptation arise from the intrinsic properties of individual neurons as opposed to their connectivity within a neuronal network? This question underpins the theme of two research articles in this Research Topic. Using *in vitro* whole-cell electrophysiology from neurons in the auditory midbrain of embryonic birds, Malinowski et al. identified a link between the rapid adaptation behaviour of neurons and their intrinsic physiological properties but not their morphological classification. Phasically spiking neurons exhibiting strong adaptation with faster recovery were found to have higher thresholds, lower membrane resistance and lower membrane time-constants compared to tonically spiking neurons. These two populations exhibited few dendritic morphological differences. Conversely, neurons with different dendritic morphological structures (stellate vs. elongated neurons) showed no significant difference in their intrinsic properties and spiking patterns. Quiroga et al., taking a computational approach, predicted that recurrent connections in a population of orientation selective neurons can give rise, over short time scales of a few hundred ms, to attractive perceptual aftereffects instead of commonly reported repulsive aftereffects. They further employed a psychophysical paradigm, appositely designed to capture aftereffects over short time scales, in order to verify this prediction. Their results indicate how recurrent network dynamics can contribute to shape perceptual adaptation.

How does adaptation affect network dynamics and patterns of functional connectivity? Martin et al. addressed this question in a well-understood and relatively simple spinal network. Using a combination of graph theoretic and Markov analyses of cord dorsum potentials recorded from cat lumbar segments, they characterised how pharmacologically-delivered nociceptive stimulation alters functional connectivity. These alterations represent transitions between different states of synchrony in the network activity shaped by supra-spinal inputs. Adibi and Lampl identified different profiles of stimulus-dependent synchrony in sensory thalamus versus cortex over various levels of adaptation, with sensory cortex maintaining its pattern of synchrony in spiking activity across spontaneous and adapted cortical states. Zarei et al. quantified the temporal coupling of spike times to phasic field potentials as a function of stimulus (frequency of auditory signal) in rat auditory cortex in adapted and non-adapted conditions. They observed that phase coupling is tuned to stimulus frequency and reduces with adaptation. Collectively, the findings in these studies illustrate the diversity of the effects adaptation generates at the network level.

What neuronal computations underlie sensory adaptation? Latimer and Fairhall investigated this question using a simple statistical model of spiking neurons based on an inhomogeneous Poisson process with a rate that is a linear combination of the stimulus and recent spiking history. This model explains how adaptation can change the gain of neuronal responses based on the variance of the stimulus as well as capturing observations of adaptation across multiple time scales.

Over the last two decades, accumulated evidence has revealed adaptation to high-level features and complex elements of perception including facial signals such as emotion and eye gaze. Gwinn et al. show electro-physiological and perceptual effects of adaptation to the variance of different facial configurations, suggesting that adaptation shapes face perception not only based on the average characteristics of the faces we observe, but also based on the range of faces. Whereas, adaptation typically tends to bias perception away from the adaptor (in the form of negative or repulsive after-effects), the phenomenon of priming indicates that the processing of a repeated stimulus can be facilitated. In a review of the literature on face adaptation and face priming, Mueller et al. draw upon these two phenomena to gain insight into the nature of the perceptual “space” within which faces are represented for recognition. As we ascend the visual hierarchy the ability of attention to modulate processing tends to increase (Reynolds and Chelazzi, 2004). Thus, the extent to which adaptation to a particular stimulus attribute depends on the amount of attention to the adaptor can be used to infer the level(s) of processing involved. Tonelli et al. show that the amount of attention a subject pays to an adapting stimulus does not affect adaptation to the size of a visual stimulus, consistent with previous findings suggesting that local gain control in primary visual cortex mediates size adaptation (Murray et al., 2006; Fang et al., 2008; Sperandio et al., 2012; Pooresmaeili et al., 2013).

Motion is a fundamental quality of vision that can create strong adaptation effects at various stages of processing (Barlow and Hill, 1963; Hammond et al., 1985; Petersen et al., 1985; Huk et al., 2001) for review see (Anstis et al., 1998; Mather et al., 1998). Despite diverse evolutionary constraints across different species, from insects and cephalopods to vertebrates, visual systems have converged to provide motion detection by conceptually similar mechanisms to detect local and global (whole retinal image) motion crucial to maintaining accurate vision during eye and/or body movement (Clifford and Ibbotson, 2002). Matsumoto and Tachibana studied how motion simulating the jitter of the retinal image during fixation affects the receptive field (RF) of retinal ganglion cells. Their study revealed that global motion leads to elongated RFs and temporal sharpening of evoked responses in a group of ganglion cells with phasic response profile. Pharmacological manipulations identified two plausible mechanisms underlying these RF alterations: electrical coupling between bipolar cell dendrites, and presynaptic inhibition by amacrine cells. Li et al. developed an innovative stimulus paradigm to disentangle local motion adaptation at the level of elementary motion detectors in blowflies from global motion adaptation affecting wide-field neurons that pool the output of elementary detectors. They found that global motion adaptation is strongly direction dependent, while local motion adaptation is largely direction independent, potentially leading to a robust representation of the direction of local motion independent of global motion direction.

Adaptation is a hallmark not only of vision but also of tactile perception and has considerable behavioural relevance to animals such as rats and mice whose primary sensory modality is their whisker-mediated tactile system (Adibi, 2019). Adibi and Lampl provide a thorough review of the physiology of neuronal adaptation along the somatosensory pathways from receptors to brainstem, sensory thalamus and cortex, outlining distinctive features of sensory adaptation in the rodent whisker-mediated tactile system. They further comment on the underlying mechanisms of adaptation and its functional roles, suggesting a diverse range of functions including shifting the operating mode of cortical computation along the continuum of coincidence detection and temporal integration, optimising receptive fields, performing noise reduction, enhancing salience detection, and frequency-domain filtering properties, depending on the dynamics of stimulation in the environment. Using intracellular recording of neurons in rat barrel cortex, Katz and Lampl showed that in most layer 2/3 neurons, similar to layer 4 neurons, adaptation is whisker-specific, despite their multi-whisker receptive fields. This finding indicates that multi-whisker receptive fields in layer 2/3 mainly emerge from intracortical horizontal connections with neighboring barrels rather than being inherited from layer 4 neurons. This allows high responsiveness in complex environments.

A consequence of statistical regularities in the environment is increased *predictability* of specific stimuli. Adibi and Lampl suggest that reduced responsiveness of sensory neurons to repetitive stimuli over time may represent a reduction in *prediction error* (also a hallmark of predictive coding theory) and provides a mechanism for enhanced detection of deviant stimuli from the background (for further review refer to Pazo-Alvarez et al., 2003; Winkler and Czigler, 2012; Grimm et al., 2016; Carbajal and Malmierca, 2018). Ross and Hamm summarised recent evidence on the potential neuronal mechanisms underlying adaptation and deviance detection, particularly in the context of “mismatch negativity” (MMN), a negative event-related scalp potential recorded at about 150 ms post-stimulus onset in response to deviant stimuli compared to redundant stimuli in an oddball paradigm. To dissociate stimulus repetition suppression (adaptation) and expectation suppression (prediction) of fMRI responses in the human brain, Amado et al. used pairs of face stimuli presented with inter-stimulus intervals (ISIs) between 0.5 and 3.75 s, where the gender of the first stimulus indicated either repetition of the same face or predicted a novel face. They found repetition and expectation suppression effects in face-sensitive visual cortex that were both independent of the length of the ISI.

Our hope is these brief introductory remarks will spark the interest of readers to delve deeper into a field of research that, despite a long history and a well-established body of literature, still continues to flourish and yield new insights into the mechanisms and functions of a key aspect of sensory processing and perception.

## Author Contributions

MA: original draft. All authors edited and approved the final version.

## Conflict of Interest

The authors declare that the research was conducted in the absence of any commercial or financial relationships that could be construed as a potential conflict of interest.

## Publisher's Note

All claims expressed in this article are solely those of the authors and do not necessarily represent those of their affiliated organizations, or those of the publisher, the editors and the reviewers. Any product that may be evaluated in this article, or claim that may be made by its manufacturer, is not guaranteed or endorsed by the publisher.
